# Prognostic and clinicopathological significance of systemic immune-inflammation index in pancreatic cancer: a meta-analysis of 2,365 patients

**DOI:** 10.18632/aging.203449

**Published:** 2021-08-25

**Authors:** Yifang Shui, Mengquan Li, Jing Su, Mingxun Chen, Xiaobin Gu, Wenzhi Guo

**Affiliations:** 1 Department of Hepatobiliary and Pancreatic Surgery, The First Affiliated Hospital of Zhengzhou University, Zhengzhou 450052, Henan, China; 2 Henan Key Laboratory of Digestive Organ Transplantation, Zhengzhou 450052, Henan, China; 3 Open and Key Laboratory of Hepatobiliary and Pancreatic Surgery and Digestive Organ Transplantation at Henan Universities, Zhengzhou 450052, Henan, China; 4 Zhengzhou Key Laboratory of Hepatobiliary and Pancreatic Diseases and Organ Transplantation, Zhengzhou 450052, Henan, China; 5 Department of Breast Disease Surgery, The First Affiliated Hospital of Zhengzhou University, Zhengzhou 450052, Henan, China; 6 Department of Hepatobiliary and Pancreatic Surgery, Zhengzhou Central Hospital Affiliated to Zhengzhou University, Zhengzhou 450007, Henan, China; 7 Department of Radiation Oncology, The First Affiliated Hospital of Zhengzhou University, Zhengzhou 450052, Henan, China

**Keywords:** meta-analysis, pancreatic cancer, prognosis, biomarker, systemic immune-inflammation index

## Abstract

The prognostic value of the systemic immune-inflammation index (SII) in patients with pancreatic cancer is conflicting according to previous investigations. Therefore, we performed a meta-analysis to explore the association between SII and pancreatic cancer prognosis. Electronic databases were searched for studies exploring the association of SII with prognostic outcomes in pancreatic cancer. The endpoints were overall survival (OS), disease-free survival (DFS), recurrence-free survival (RFS), progression-free survival (PFS), cancer-specific survival (CSS), and clinicopathological parameters. The prognostic value of SII was estimated by hazard ratio (HR) or odds ratio (OR) with a 95% confidence interval (CI). Nine studies containing 11 cohorts with 2,365 subjects in total were included in this meta-analysis. Elevated SII was associated with poor OS (HR=1.50, 95% CI=1.15–1.96, p=0.002), RFS/PFS/DFS (HR=1.52, 95% CI=1.01–2.28, p=0.045), and CSS (HR=2.60, 95% CI=1.65–4.09, p < 0.001) in patients with pancreatic cancer. Additionally, there was no significant association between SII and other parameters in pancreatic cancer such as sex, tumor location, lymph node metastasis, tumor-node-metastasis stage, vascular invasion, and grade. This meta-analysis suggested that elevated SII was a significant prognostic marker for short-term and long-term survival outcomes in patients with pancreatic cancer.

## INTRODUCTION

Pancreatic cancer is a highly aggressive malignant tumor and the seventh leading cause of cancer-related deaths worldwide [1, 2]. In 2018, 458,918 new cases and 432,242 deaths due to pancreatic cancer occurred globally [1]. A lack of reliable early biomarkers causes 85% of patients to be diagnosed with metastatic or locally advanced disease [3]. The prognosis of pancreatic cancer is poor with a 5-year survival rate of less than 5% [4]. Surgical resection is the only curative approach for pancreatic cancer and is feasible in 15% of the cases [4]. However, even for patients with operable pancreatic cancer, the 5-year survival rate is only 18%–24% [5]. One of the major reasons for the poor prognosis is the lack of effective biomarkers [4]; therefore, identification of novel prognostic markers is pivotal for better management of patients with pancreatic cancer.

Various studies have shown that systemic inflammation plays an important role in cancer progression [6]. Systemic inflammatory responses are involved in the initiation, promotion, and metastasis of cancer cells [7]. In the recent years, the systemic immune-inflammation index (SII), which is calculated as platelet count × neutrophils/lymphocytes, has been reported as a noninvasive prognostic marker for various solid tumors [8, 9]. The prognostic value of SII in patients with pancreatic cancer has also been explored by many researchers [10–15]; however, the results are conflicting. For example, elevated SII in patients with pancreatic cancer was found to be associated with poor survival outcomes in some studies [14, 15] and favorable prognosis in other studies [11]. Therefore, in this study, we collected the literature published in this area of study and conducted a meta-analysis. We hypothesized that elevated SII could be a significant prognostic factor for patients with pancreatic cancer. We aimed to clarify the prognostic impact of SII on pancreatic cancer and analyze the correlation between SII and the clinicopathological features of pancreatic cancer.

## RESULTS

### Literature search and study characteristics

The initial literature search identified 64 studies, out of which, 23 studies were selected after eliminating the duplicate records. Following the examination of titles and abstracts, 11 studies were excluded, and the entire text of the remaining 12 studies was examined. Subsequently, six studies were excluded for the following reasons: two studies did not provide survival outcomes, two studies did not identify the cut-off value of SII, one study did not provide usable data for analysis, and one study was a meeting abstract. Following an updated literature search, three additional studies [16–18] were included in the meta-analysis.

**Figure 1:**
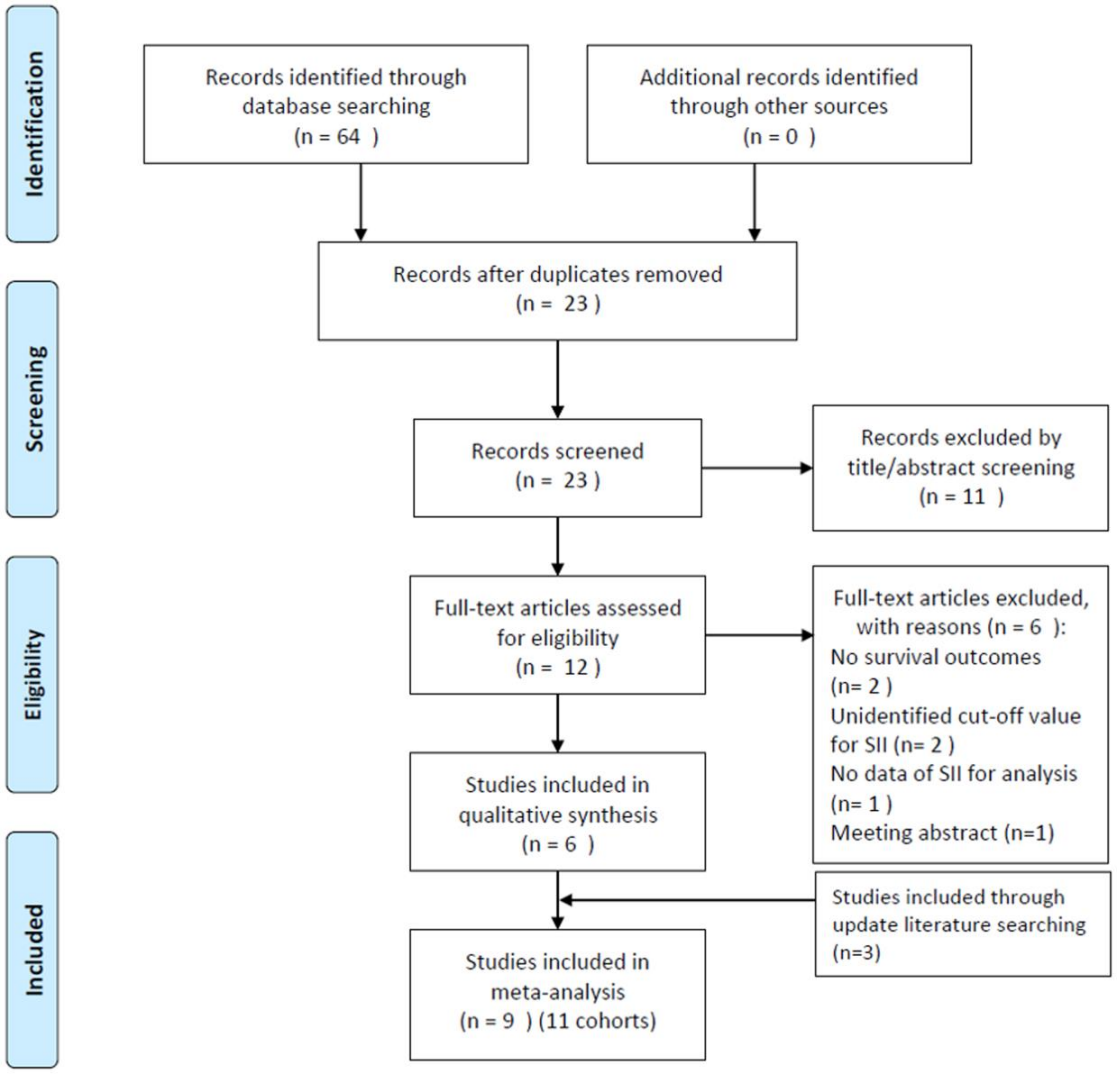
Flow diagram showing the selection of literature for the meta-analysis.

At the end of the selection process, nine studies [10–18] were included in this meta-analysis (Figure 1). In the studies by Aziz et al. [10] and Zhang et al. [14] studies, two independent cohorts were recruited in each study, which were labeled as cohorts Aziz, M. H. (B) [10], Aziz, M. H. (B) [10], Zhang, K. (A) [14], and Zhang, K. (B) [14]. Therefore, nine studies containing 11 cohorts were included in the meta-analysis. The basic characteristics of the included studies are listed in Supplementary Table 1; these studies were published from 2019 to 2021. The 11 cohort studies were conducted in China (n=4) [12, 14, 15, 17, 18], the Netherlands (n=2) [10], Austria (n=1) [11], Italy (n=1) [16], and the United States of America (USA) (n=1) [13]. The sample size ranged from 27 to 420, and the total sample size was 2,365. Nine cohort studies [10–14, 16, 17] were published in English and two [15, 18] were published in Chinese. Regarding the study design, nine cohorts [10, 12–18] were retrospective studies and two [11, 12] were prospective studies. The cut-off value of SII ranged from 440 to 1200. Nine cohorts [11–18] reported the prognostic role of SII in OS, six cohorts [10–12, 16, 17] reported an association between SII and RFS/PFS/DFS, and two cohorts reported CSS [10]. The Newcastle-Ottawa Scale (NOS) scores ranged from 6 to 8, indicating that all the studies included were of high quality.

### SII and OS in pancreatic cancer

**Figure 2:**
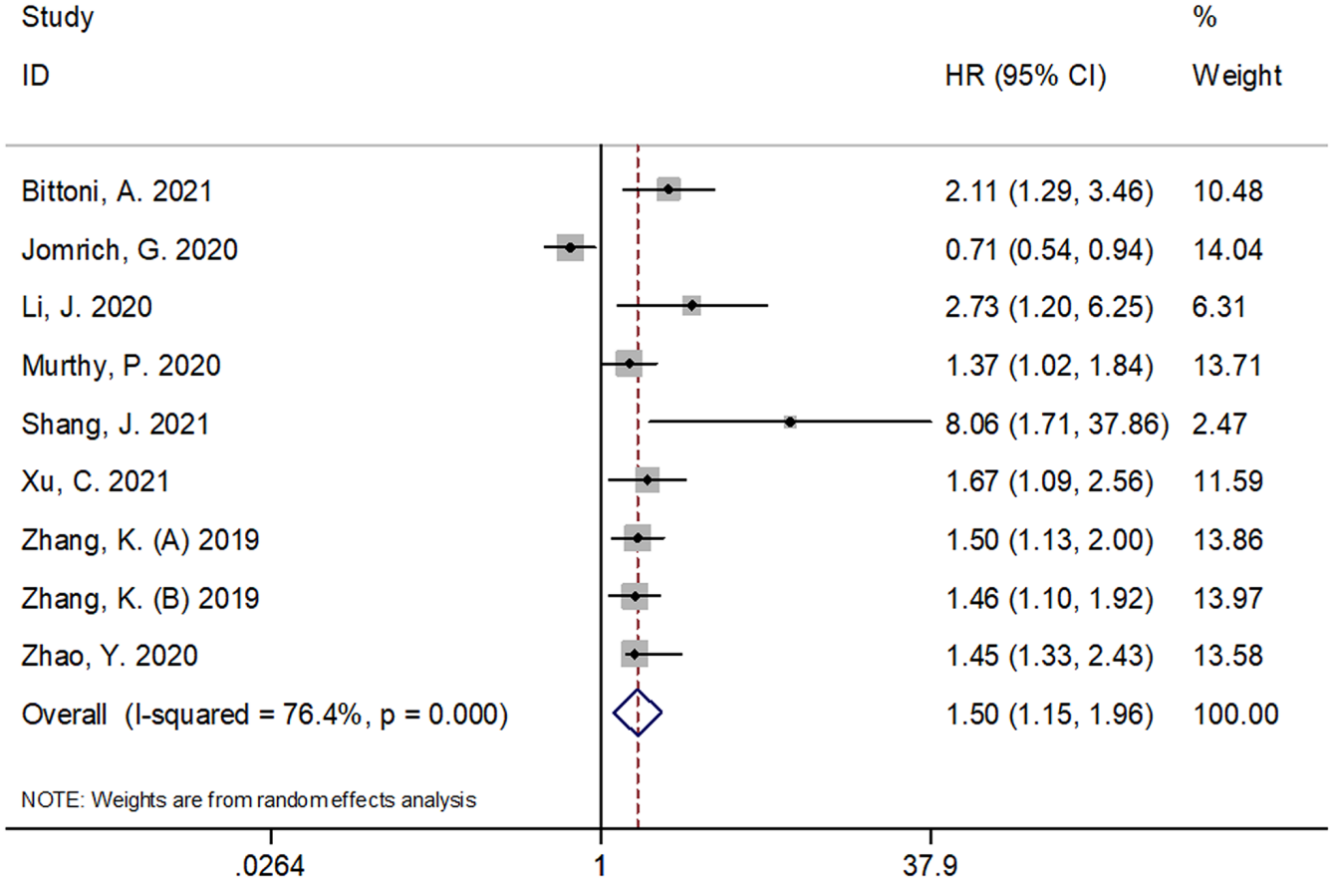
**Forest plot reflecting the association between SII and OS in pancreatic cancer.** A random-effects model (REM) was used because of significant heterogeneity (*I*^2^ = 76.4%, P < 0.001). The pooled HR and 95%CI are: HR=1.50, 95%CI=1.15-1.96, p=0.002. SII= systemic immune-inflammation index, OS=overall survival.

A total of nine cohorts with 1,775 subjects [11–18] reported an association between SII and OS in pancreatic cancer. A random-effects model (REM) was used because of significant heterogeneity (*I*^2^ = 76.4%, P < 0.001). The pooled results were as follows: HR=1.50, 95% CI =1.15–1.96, p=0.002 (Figure 2; Table 1), suggesting that SII was not a significant prognostic marker for OS. In the subgroup analysis of these patients with pancreatic cancer, the results demonstrated that SII was a significant prognostic factor for OS in patients of Asian ethnicity, in stage III-IV/recurrent disease, in retrospective studies, and with an SII cut-off value ≥ 900 (Table 1).

**Table 1:** Subgroup analysis of the prognostic role of SII for OS, RFS/PFS/DFS, and CSS for patients with pancreatic cancer.

**Subgroups**	**Studies (n)**	**Patients (n)**	**HR (95%CI)**	**p**	**Effects model**	**Heterogeneity**	
						***I*^2^(%)**	**Ph**
OS							
Total	9	1,775	1.50(1.15-1.96)	0.002	REM	76.4	<0.001
Ethnicity						
Caucasian	3	977	1.24(0.69-2.24)	0.477	REM	89.1	<0.001
Asian	6	798	1.55(1.33-1.80)	<0.001	FEM	25.6	0.243
Sample size						
<200	5	576	1.59(1.33-1.91)	<0.001	FEM	37.8	0.169
≥200	4	1,199	1.28(0.84-1.97)	0.251	REM	86.0	<0.001
TNM stage						
I-III	4	973	1.22(0.83-1.81)	0.311	REM	83.5	<0.001
III-IV/recurrent	5	802	1.63(1.36-1.95)	<0.001	FEM	47.4	0.107
SII cut-off value						
<900	6	987	1.47(1.00-2.14)	0.048	REM	82.6	<0.001
≥900	3	788	1.57(1.26-1.95)	<0.001	FEM	12.8	0.318
Study design						
Retrospective	7	1,424	1.52(1.34-1.74)	<0.001	FEM	14.6	0.319
Prospective	2	351	1.32(0.36-4.89)	0.680	REM	89.1	0.002
Cut-off determination						
ROC analysis	5	802	1.74(0.96-3.14)	0.068	REM	86.6	<0.001
X-tile/other	4	973	1.47(1.26-1.72)	<0.001	FEM	0	0.895
RFS/PFS/DFS						
Total	6	1,297	1.52(1.01-2.28)	0.045	REM	72.5	0.003
Ethnicity						
Caucasian	4	1,148	1.27(0.83-1.93)	0.267	REM	73.9	0.009
Asian	2	149	2.76(1.45-5.25)	0.002	FEM	0	0.944
Sample size						
<200	3	170	1.96(1.26-3.05)	0.003	FEM	2.1	0.360
≥200	3	978	1.23(0.73-2.07)	0.429	REM	81.5	0.005
TNM stage						
I-III	3	914	1.19(0.69-2.04)	0.527	REM	72.8	0.025
III-IV/recurrent	3	234	1.73(1.28-2.35)	<0.001	FEM	22.6	0.275
SII cut-off value						
<900	3	473	1.68(0.62-4.55)	0.303	REM	83.5	0.002
≥900	3	824	1.55(1.19-2.02)	0.001	FEM	0	0.908
Study design						
Retrospective	4	946	1.61(1.24-2.08)	<0.001	FEM	0	0.685
Prospective	2	351	1.37(0.41-4.57)	0.609	REM	86.7	0.006
CSS						
Total	2	590	2.60(1.65-4.09)	<0.001	FEM	0	0.721

HR, hazards ratio; CI, confidence interval; ROC, receiver operating characteristic; OS, overall survival; RFS, recurrence-free survival; DFS, disease-free survival; PFS, progression-free survival; CSS, cancer-specific survival; TNM, Tumor- Node- Metastasis; REM, random-effects model; FEM, fixed-effects model.

### SII and RFS/PFS/DFS in pancreatic cancer

**Figure 3:**
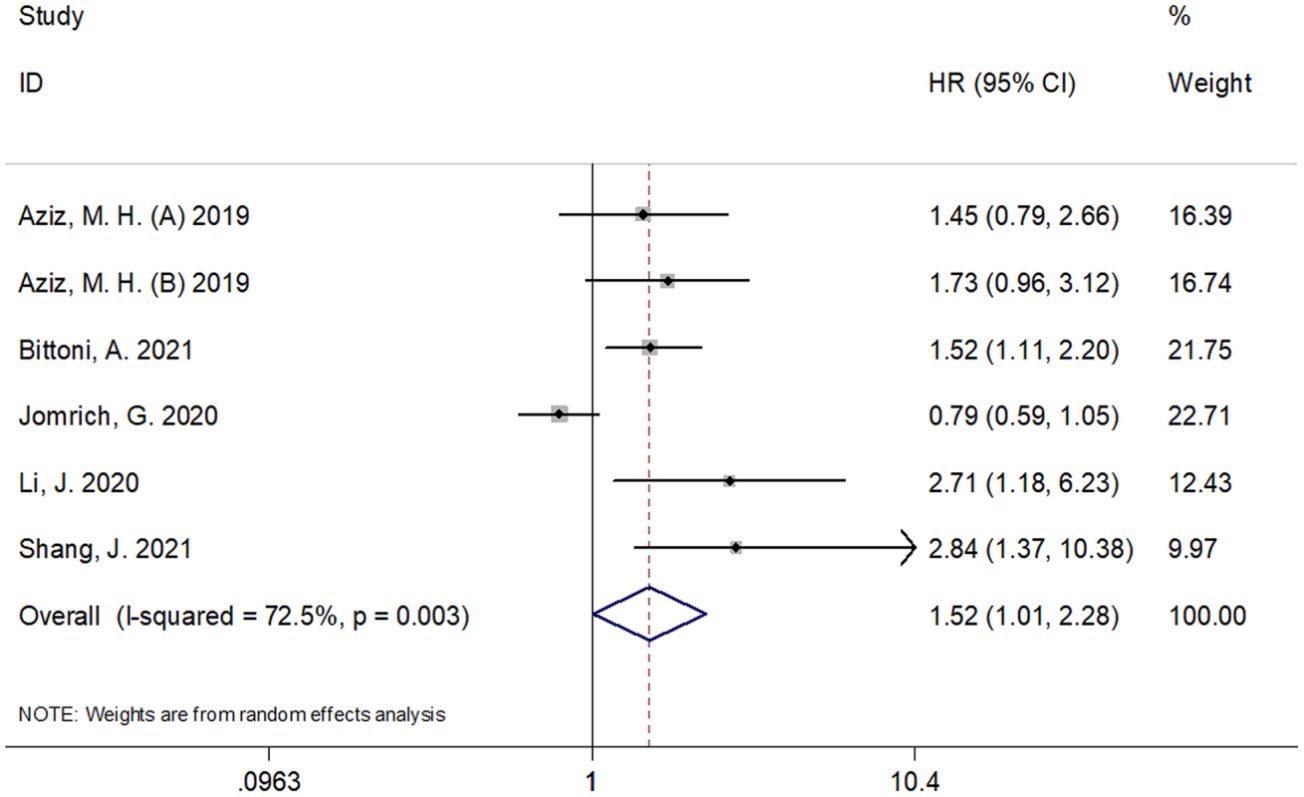
**Forest plot reflecting the association between SII and RFS/PFS/DFS in pancreatic cancer.** (HR=1.52, 95%CI=1.01-2.28, p=0.045). SII= systemic immune-inflammation index, RFS=recurrence-free survival, DFS= disease-free survival, PFS = progression-free survival.

Six cohorts, with 1,297 subjects [10–12, 16, 17], reported the prognostic value of SII for RFS/PFS/DFS. As shown in Figure 3 and Table 1, the pooled results were as follows: HR=1.52, 95% CI=1.01–2.28, p=0.045. The combined data indicated that SII was significantly associated with RFS/PFS/DFS in pancreatic cancer. Similar to the results of OS, the subgroup analysis of these patients with pancreatic cancer showed that a high SII was an indicator of poor RFS/PFS/DFS in patients of Asian ethnicity, in stage III-IV/recurrent disease, in retrospective studies, and with a SII cut-off value ≥ 900 (Table 1).

### SII and CSS in pancreatic cancer

**Figure 4:**
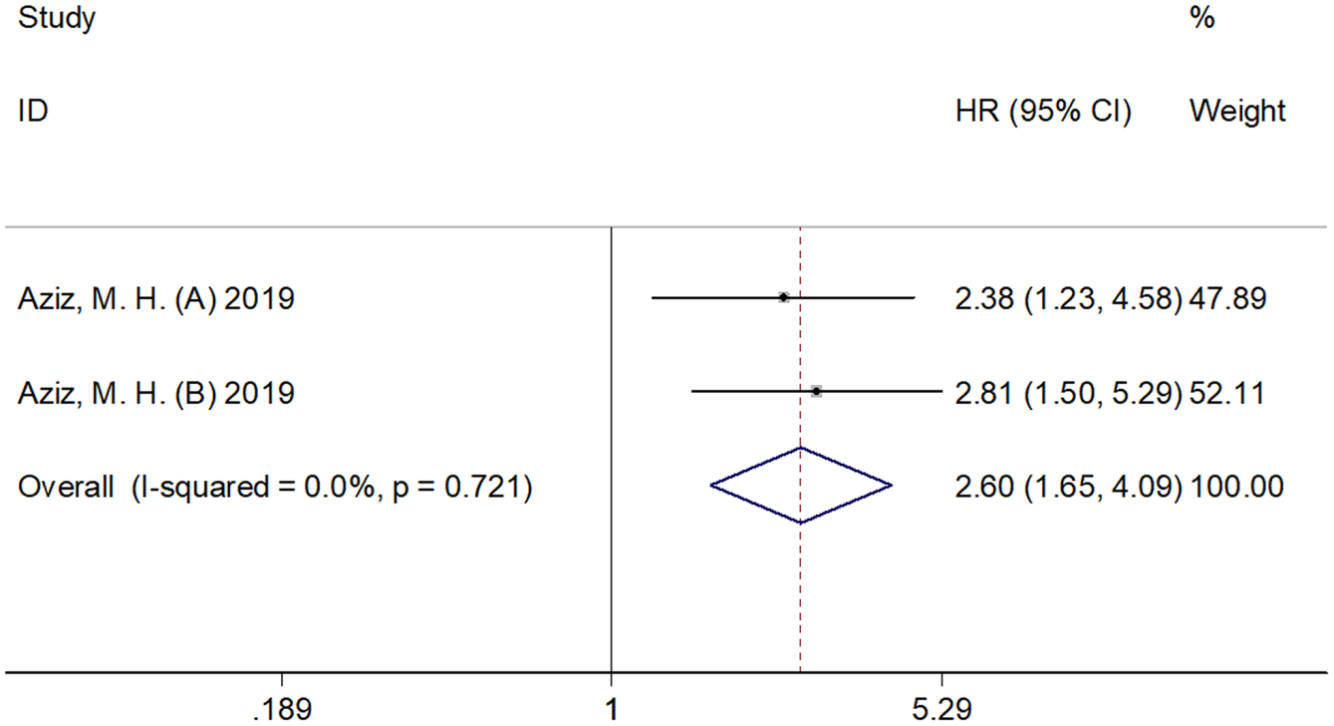
**Forest plot reflecting the association between SII and CSS in pancreatic cancer.** The combined results were: HR=2.60, 95%CI=1.65-4.09, p<0.001, which indicated that elevated SII was significantly correlated to inferior CSS in pancreatic cancer. SII= systemic immune-inflammation index, CSS= cancer-specific survival.

The association between SII and CSS was analyzed based on the data from two cohorts [10]. The pooled results were as follows: HR=2.60, 95% CI=1.65–4.09, p < 0.001 (Figure 4; Table 1), which indicated that elevated SII had significant correlation with a low CSS in pancreatic cancer. Subgroup analysis was not performed as only two cohorts were included in the analysis.

### The association between SII and clinicopathological characteristics

**Figure 5:**
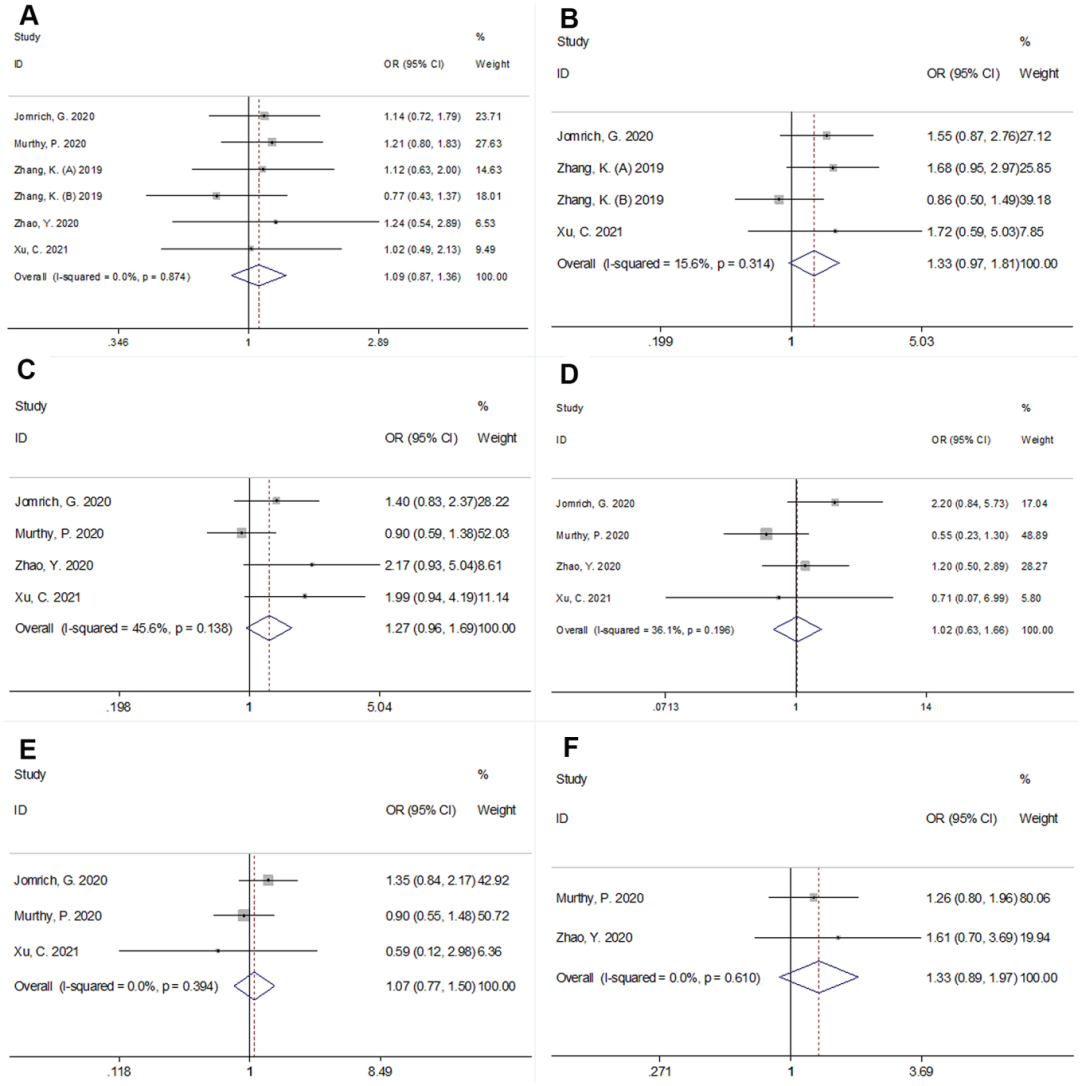
**Forest plots showing the association between SII and clinicopathological factors in pancreatic cancer.** (**A**) sex (male vs female); (**B**) tumor location (head vs body/tail); (**C**) lymph node metastasis; (**D**) Tumor-Node-Metastasis (TNM) stage (III-IV vs I-III); (**E**) grade (3-4 vs 1-2), and (**F**) vascular invasion (yes vs no).

The association between SII and clinicopathological factors was investigated based on the data from six cohorts [11, 13–15, 18]. As shown in Figure 5 and Table 2, there was no significant association between SII and sex (male vs. female) (OR=1.09, 95% CI=0.87–1.36, p=0.469), tumor location (head vs. body/tail) (OR=1.33, 95% CI=0.97–1.81, p=0.074), lymph node metastasis (yes vs. no) (OR=1.27, 95% CI=0.96–1.69, p=0.093), tumor-node-metastasis stage (III-IV vs. I-III) (OR=1.02, 95% CI=0.63–1.66, p=0.798), vascular invasion (yes vs. no) (OR=1.33, 95% CI=0.89–1.97, p=0.160), or grade (3–4 vs 1–2) (OR=1.07, 95% CI=0.77–1.50, p=0.671).

**Table 2:** Correlation between SII and clinicopathological features in patients with pancreatic cancer.

**Factors**	**Studies (n)**	**Patients (n)**	**OR (95%CI)**	**p**	**Effects model**	**Heterogeneity** ***I*^2^(%) Ph**	
Sex (male vs female)	6	1,392	1.09(0.87-1.36)	0.469	FEM	0	0.874
Tumor location (Head vs body/tail)	4	878	1.33(0.97-1.81)	0.074	FEM	15.6	0.314
Lymph node metastasis (Yes vs no)	4	973	1.27(0.96-1.69)	0.093	FEM	45.6	0.138
TNM stage (III-IV vs I-III)	4	973	1.02(0.63-1.66)	0.798	REM	56.0	0.103
Grade (3-4 vs 1-2)	3	878	1.07(0.77-1.50)	0.671	FEM	0	0.394
Vascular invasion (Yes vs no)	2	514	1.33(0.89-1.97)	0.160	FEM	0	0.610

OR, odds ratio; CI, confidence interval; TNM, Tumor- Node- Metastasis; REM, random-effects model; FEM, fixed-effects model.

### Publication bias

**Figure 6:**
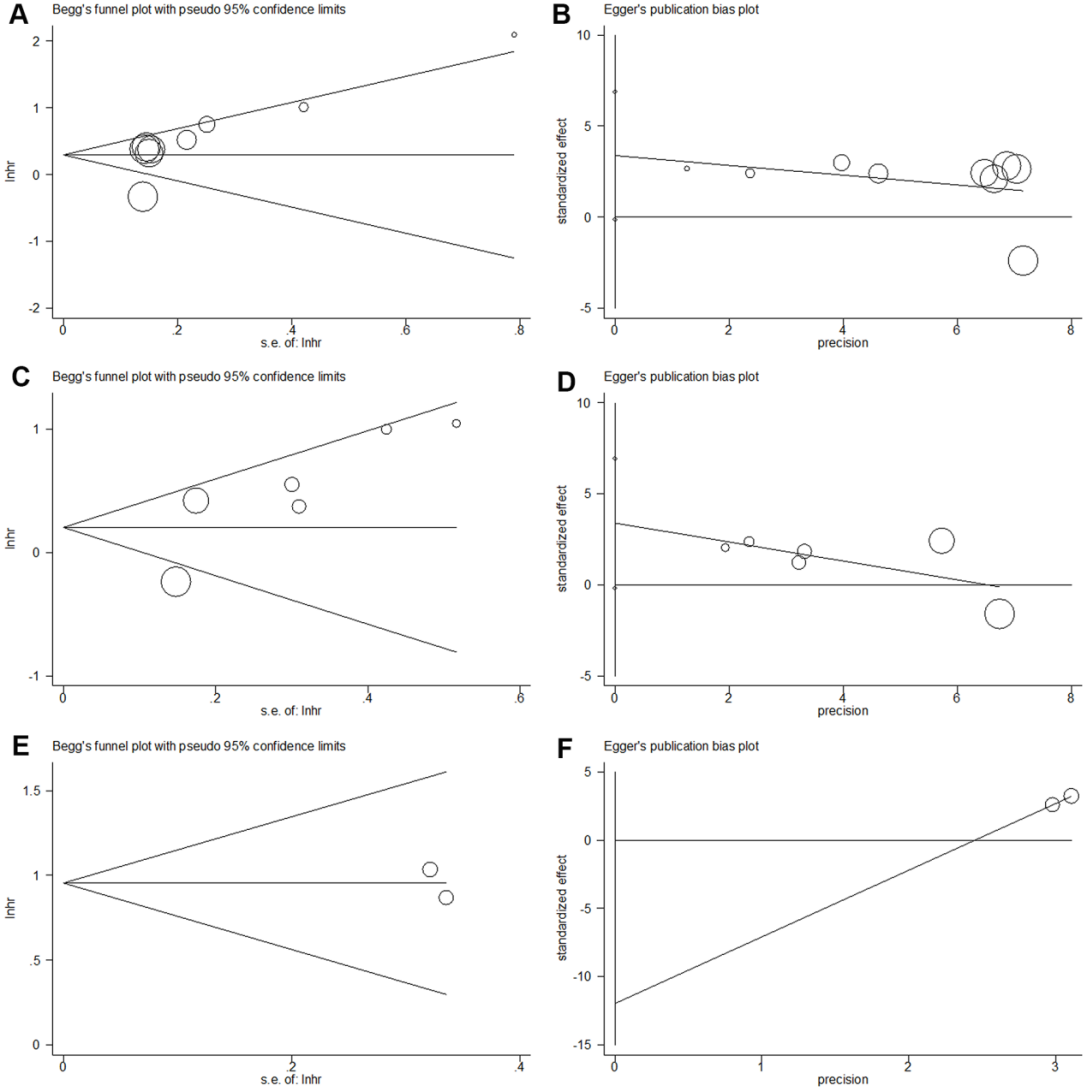
**Begg’s funnel plot and Egger’s linear regression test for publication bias in this meta-analysis.** (**A**) Begg’s funnel plot for OS (p=0.089); (**B**) Egger’s linear regression test for OS (p=0.057); (**C**) Begg’s funnel plot for RFS/PFS/DFS (p=0.260); (**D**) Egger’s linear regression test for RFS/PFS/DFS (p=0.160); (**E**) Begg’s funnel plot for CSS (p=1), and (**F**) Egger’s linear regression test for CSS (p=1).

Publication bias was analyzed using Begg’s funnel plots and Egger's regression test. As shown in Figure 6, the results indicated that there was no significant publication bias in this meta-analysis.

## DISCUSSION

The previous studies published contradictory reports on the prognostic value of SII in patients with pancreatic cancer [10–15]. In this meta-analysis of data gathered from 11 cohorts, with 2,365 subjects, we demonstrated that elevated SII was a significant prognostic factor for OS, RFS/PFS/DFS, and CSS in pancreatic cancer. The subgroup analysis indicated that elevated SII was predictive of low OS and RFS/PFS/DFS in patients of Asian ethnicity, in stage III-IV/recurrent disease, and with a SII cut-off value ≥ 900. In addition, we found that there was no significant correlation between SII and several clinicopathological features in patients with pancreatic cancer. The null association between SII and clinicopathological characteristics could have resulted because of the limited sample size in each group. In conclusion, this meta-analysis demonstrated that elevated SII was associated with poor short-term and long-term survival outcomes. Elevated SII correlated with poor long-term survival outcomes in patients of Asian ethnicity. We opine that SII is an easily available and effective biomarker which can be utilized for the prognostication of patients with pancreatic cancer in clinical practice. To the best of our knowledge, this study is the first meta-analysis to explore the prognostic value of SII in pancreatic cancer.

In the recent years, there has been accumulating evidence on the relationship between cancer progression and inflammatory response [19, 20]. A series of inflammatory biomarkers, including neutrophil/lymphocyte ratio [21, 22], platelet to lymphocyte ratio [23, 24], and SII have been proven effective for prognosis prediction in patients with cancer. SII is defined as platelet count × neutrophil count/lymphocyte count, which considers the combined effect of platelet, neutrophil, and lymphocyte counts. Elevated SII represents an increase in platelet and neutrophil counts and/or a decrease in lymphocyte count. Platelets can directly promote the growth of tumor cells by secreting various cytokines that facilitate angiogenesis and tumor progression [25]. In addition, tumor-infiltrating neutrophils have been shown to play an important role in tumor progression [26]. In contrast, lymphocytes play a major role in suppressing cancer cell proliferation and migration [27]. Tumor-infiltrating lymphocytes (TILs) can exert anti-tumor activity by inducing cytotoxic cell death and cytokine production [28]. Therefore, elevated SII being an indicator of poor prognosis is based on the diverse roles of platelets, neutrophils, and TILs in tumor biology.

Recently, many meta-analyses have explored the prognostic effect of SII in various solid tumors [29–33]. In a study on patients with breast cancer, Zhang et al. showed that an elevated SII predicted poor survival outcomes and was associated with clinicopathological features that indicated tumor progression [33]. In another recent meta-analysis, which included 3,074 patients, the researchers indicated that SII might be a promising noninvasive predictor in patients with urologic cancers [30]. Wang’s meta-analysis of 2,796 patients demonstrated that elevated SII was a poor prognostic factor for patients with hepatocellular carcinoma [32]. Our previous meta-analysis showed that elevated SII levels predicted poor prognosis in patients with colorectal cancer. In addition, elevated SII levels were also associated with clinical factors, implying higher malignancy of the disease [29]. In compliance with the results of the previous meta-analysis, the present meta-analysis showed that an elevated SII was associated with poor CSS in pancreatic cancer. Furthermore, the results also indicated that the SII is an effective marker for OS and DFS in Asian patients, which suggests that SII may be more applicable in patients of Asian ethnicity. However, the data indicated a non-significant association between SII and the clinicopathological factors of pancreatic cancer. This may be due to the relatively small sample size. Notably, the meta-analysis did not include unpublished studies and conference abstracts for the following reasons: Firstly, the unpublished literature and conference abstracts did not present full text including the results and the process of data analysis. Secondly, the full-text articles published in peer-reviewed journals were preferred because of their high quality and reliable results. Thirdly, publication bias tests did not detect publication bias or selection bias in this meta-analysis.

Nevertheless, our study has several limitations. Firstly, the sample size was relatively small. Although 11 cohorts were included, the total sample size was 2,365. The relatively small sample size might have resulted in a null association between SII and clinicopathological factors of pancreatic cancer. Secondly, this meta-analysis was limited to studies published in English and Chinese as the publications in other languages were unavailable. This could have led to a possible selection bias in this meta-analysis. Thirdly, the cut-off values of SII vary among the studies included, which may significantly contribute to substantial heterogeneity in this meta-analysis. Therefore, a uniform cut-off value for SII is needed in further studies.

In summary, this meta-analysis suggests that an elevated SII is a significant prognostic marker for short-term and long-term survival outcomes. The SII has a significant prognostic role in Asian patients with pancreatic cancer. Therefore, we suggest that SII be employed as an effective biomarker for the prognosis of patients with pancreatic cancer in clinical practice.

## MATERIALS AND METHODS

### Literature search strategy

This meta-analysis was performed in accordance with the Preferred Reporting Items for Systematic Reviews and Meta-Analyses statement [34]. The electronic databases PubMed, Embase, Web of Science, Cochrane Library, China National Knowledge Infrastructure, and China Wanfang databases were searched systematically. The search strategy was as follows: (“systemic immune-inflammation index” or “SII”) and (“pancreatic cancer” or “pancreatic neoplasms” or “pancreatic adenocarcinoma” or “pancreatic tumor”). The search duration was from inception to March 25, 2021. There were no language restrictions. In addition, the references of the included publications and reviews were manually checked for potentially eligible studies.

### Inclusion and exclusion criteria

Two investigators (Y.S. and M.L.) independently performed the literature search, and any disagreements were resolved by consensus. The selection criteria were established based on previous meta-analyses of the SII [29, 32, 33]. The inclusion criteria were as follows: (a) diagnosis of pancreatic cancer was pathologically confirmed; (b) SII was defined as the neutrophil count × platelet count/lymphocyte count; (c) patients who did not have active infections, inflammatory conditions, or comorbid diseases before blood examination; (d) studies exploring the association between SII and survival outcomes in pancreatic cancer with hazard ratios (HRs) and 95% confidence intervals (CIs); (e) the cut-off value of SII was provided; and (f) articles published in English or Chinese. The exclusion criteria were as follows: (a) meeting abstracts, reviews, letters, case reports, and comments; (b) animal studies; and (c) insufficient information available for data analysis. Outcomes of interest included overall survival (OS), recurrence-free survival (RFS), progression-free survival (PFS), disease-free survival (DFS), and cancer-specific survival (CSS). The primary endpoint was the OS, and the secondary endpoints were the RFS/PFS/DFS and CSS.

### Data extraction and quality assessment

Two investigators (Y.S. and M.C.) independently extracted data from the included studies, and discrepancies were resolved by discussion with a third investigator (X.G.). The following information was extracted: name of the first author, year of publication, country, sample size, age, histological type, tumor stage, treatment, study period, cut-off value of SII, cut-off determination method, study design, follow-up, survival endpoints, survival analysis, and the HRs and 95% CIs of survival outcomes. The quality of the included studies was evaluated using the NOS [35], which evaluates the quality of the study in three aspects: selection, comparability, and exposure. The NOS scores range from 0 to 9. Studies with NOS score of 6 or more were regarded as high-quality studies.

### Statistical analysis

The HRs and 95% CIs were used to evaluate the prognostic role of the SII for OS, RFS/PFS/DFS, and CSS in pancreatic cancer. The heterogeneity among studies was assessed using the Cochran’s Q test and I^2^ statistics. In the presence of significant heterogeneity (*I*^2^ > 50% and/or P < 0.10), REM was used to combine the HRs and 95% CIs. Otherwise, a fixed-effects model (FEM) was adopted. Subgroup analysis was conducted to explore the sources of heterogeneity. The association between SII and clinicopathological factors was evaluated using odds ratios and 95% CIs. Publication bias was estimated using the Begg’s test and Egger’s test. Statistical significance was set at p < 0.05. All the statistical analyses were performed using Stata version 12.0 (Stata Corporation, College Station, TX, USA).

## Supplementary Material

Supplementary Table 1
