# Supplementary material for: Prognostic and clinicopathological significance of systemic immune-inflammation index in pancreatic cancer: a meta-analysis of 2,365 patients
Source: Aging (Albany NY). 2021 Aug 25;13(16):20585–97. doi: 10.18632/aging.203449 (PMC8436945; doi:10.18632/aging.203449)
Supplement: Supplementary Table 1 [file aging-13-203449-s001.docx]

**Supplementary Table 1. Baseline characteristics of included studies in this meta-analysis.**

| Study | Year | Country | Sample size | Sex  (M/F) | Age  Median (range) | Histology type | TNM stage | Treatment | Cut-off value | Cut-off determination method | Study design | Follow-up median (months) | Survival outcomes | Study center | Ethnicity | NOS score |
| --- | --- | --- | --- | --- | --- | --- | --- | --- | --- | --- | --- | --- | --- | --- | --- | --- |
| Aziz, M. H. (A) | 2019 | the Netherlands | 170 | 96/74 | 65.5 (35.5-82.4) | PDAC | I-III | Neoadjuvant chemotherapy + surgery | 900 | ROC analysis | Retrospective | 43.3 | RFS, CSS | Multi-center | Caucasian | 7 |
| Aziz, M. H. (B) | 2019 | the Netherlands | 420 | 202/218 | 65.6(36.6-84.7) | PDAC | I-III | Neoadjuvant chemotherapy + surgery | 900 | ROC analysis | Retrospective | 44.8 | RFS, CSS | Multi-center | Caucasian | 7 |
| Bittoni, A. | 2021 | Italy | 234 | 131/103 | 67(31-85) | PDAC | III-IV | Chemotherapy | 1200 | ROC analysis | Retrospective | 29.2 | OS, PFS | Single center | Caucasian | 7 |
| Jomrich, G. | 2020 | Austria | 324 | 169/155 | 68.5(35.9-92.3) | PDAC | I-III | Neoadjuvant chemotherapy + surgery | 873 | ROC analysis | Prospective | NA | OS, DFS | Single center | Caucasian | 8 |
| Li, J. | 2020 | China | 27 | 18/9 | 58(43-81) | PDAC | Recurrent | Radiotherapy | 821.9 | ROC analysis | Prospective | To Dec 2019 | OS, PFS | Single center | Asian | 8 |
| Murthy, P. | 2020 | USA | 419 | 210/209 | 65.17 | PDAC | I-III | Neoadjuvant chemotherapy + surgery | 900 | Harrell’s C and Somers’ D statistics | Retrospective | To Jan 2019 | OS | Single center | Caucasian | 7 |
| Shang, J. | 2021 | China | 122 | 87/35 | 56(33-85) | PDAC | III-IV | Immunotherapy | 566 | ROC analysis | Retrospective | NA | OS, PFS | Single center | Asian | 8 |
| Xu, C. | 2021 | China | 135 | 78/57 | 56.6(28-76) | PDAC | I-III | Surgery | 900 | Literature | Retrospective | To Dec 2019 | OS | Single center | Asian | 7 |
| Zhang, K. (A) | 2019 | China | 197 | 122/75 | ≤60：95  >60: 102 | PDAC | III-IV | Mixed | 440 | X-tile software | Retrospective | To Dec 2016 | OS | Single center | Asian | 7 |
| Zhang, K. (B) | 2019 | China | 222 | 147/75 | ≤60: 105  >60: 117 | PDAC | III-IV | Mixed | 440 | X-tile software | Retrospective | To Dec 2017 | OS | Single center | Asian | 7 |
| Zhao, Y. | 2020 | China | 95 | 56/39 | <70: 53  ≥70: 42 | PDAC | I-III | Neoadjuvant chemotherapy + surgery | 885 | ROC analysis | Retrospective | 16 | OS | Single center | Asian | 6 |

PDAC: pancreatic ductal adenocarcinoma; ROC: receiver operating characteristic; OS: overall survival; RFS: recurrence-free survival; DFS: disease-free survival; PFS: progression-free survival; CSS: cancer-specific survival; TNM: Tumor- Node- Metastasis; NOS: Newcastle-Ottawa Scale.
